# *Saksenaea oblongispora* Rhinosinusitis in Advanced HIV: A Rare and Lethal Mucormycosis

**DOI:** 10.1155/crip/3227863

**Published:** 2025-08-17

**Authors:** Bonita van der Westhuizen, Liska Budding, Christie Esterhuysen, Samantha Potgieter

**Affiliations:** ^1^Department of Medical Microbiology, University of the Free State Faculty of Health Sciences, Bloemfontein, South Africa; ^2^National Health Laboratory Service, Universitas Academic Hospital, Bloemfontein, South Africa; ^3^Department of Anatomical Pathology, University of the Free State Faculty of Health Sciences, Bloemfontein, South Africa; ^4^Department of Internal Medicine, University of the Free State Faculty of Health Sciences, Bloemfontein, South Africa

**Keywords:** immunocompromised, Mucorales, mucormycosis, *S. oblongispora*, *Saksenaea*

## Abstract

Mucormycosis is a severe invasive infection caused by the Mucorales fungi. The most frequently implicated genera are *Rhizopus* species, *Mucor* species, and *Lichtheimia* species. These fungi do not typically cause infections in immunocompetent individuals. Risk factors include diabetes mellitus, malignancies, transplant recipients, and current or past COVID-19 infection. Mucorales have also been linked to outbreaks in healthcare settings and following natural disasters. We describe a case of rapidly progressing rhinosinusitis in a patient with advanced HIV infection due to *Saksenaea oblongispora*, a rare cause of mucormycosis that, in contrast to the other Mucorales, primarily affects immunocompetent hosts following traumatic inoculation. A 32-year-old male patient presented with right-sided facial swelling. His clinical condition deteriorated rapidly. Biopsies and computerized tomography (CT) of the brain and sinuses were performed. Tuberculosis and bacterial workups were negative. Histological examination showed thick-walled angioinvasive fungal elements. Fungal cultures were positive. Molecular testing identified the organism as *S. oblongispora*. Due to his rapid deterioration, he neither underwent surgical intervention nor received any antifungal therapy and subsequently demised. This is the first case of *S. oblongispora* infection described in sub-Saharan Africa and in the setting of HIV. Infection by this fungus accounts for approximately 3% of human mucormycosis cases. *S. oblongispora*-associated rhinosinusitis is extremely uncommon and has been associated with rapid progression with high morbidity and mortality. A combination of different testing platforms was required to make a diagnosis. This case emphasizes the challenge of diagnosing invasive mold infections timeously. A high index of suspicion, combined with a multidisciplinary diagnostic and treatment approach, is essential for the management of these infections.

## 1. Introduction

Mucormycosis is a rare but severe invasive infection caused by fungi from the Mucorales order, with the most frequently implicated genera being *Rhizopus* species (spp.), *Mucor* spp., and *Lichtheimia* spp. [1]. These fungi are commonly found in the environment and typically do not cause infections in individuals with healthy immune systems [1–3]. Mucormycosis primarily affects those with underlying health conditions such as diabetes mellitus, malignancies, organ or stem cell transplants, and, more recently, current or past COVID-19 infections [1, 2, 4]. Mucorales fungi have also been linked to outbreaks in healthcare settings and following natural disasters [1–5].

In this report, we describe a case of rapidly progressing rhinosinusitis in a patient with advanced human immunodeficiency virus (HIV) infection due to *Saksenaea oblongispora*, a rare cause of mucormycosis that, contrary to other Mucorales, primarily affects immunocompetent hosts following traumatic inoculation [2–5]. Mucormycosis caused by this pathogen has not been described in sub-Saharan Africa or in the setting of HIV infection.

## 2. Case Presentation

### 2.1. History and Clinical Presentation

A 32-year-old male patient was admitted with right-sided facial swelling. The patient was HIV-positive on antiretroviral therapy (ART), together with trimethoprim–sulfamethoxazole (TMX) prophylaxis. Additionally, the patient had hypertension for which he was also receiving treatment. The patient's facial swelling rapidly progressed, with extension of redness and swelling observed daily. Four days after admission, he underwent a computerized tomography (CT) scan, and tissue biopsies were collected. He had not received antifungal therapy or any surgical debridement procedure. The patient died 3 days later, 7 days after admission.

### 2.2. Treatment Received

The patient received the following treatments:

1. Zidovudine 300 mg twice a day (BD) per os (po)
2. Lamivudine 150 mg BD po
3. Lopinavir/ritonavir 400 mg/100 mg BD po
4. TMX 160/800 mg once daily (OD) po
5. Amlodipine 5 mg OD po
6. Enalapril 10 mg OD po

### 2.3. Laboratory Results

Laboratory investigations revealed the following:

• The patient had a CD4 count of 50 cells/*μ*L (no HIV viral load was documented).
• The serum cryptococcal antigen test was negative.
• The C-reactive protein (CRP) count was 253 g/dL on presentation.
• A full blood count revealed a mild normochromic normocytic anemia with thrombocytosis and rouleaux formation.
• Kidney and liver function tests were essentially normal.

### 2.4. Radiological Examinations

A CT scan was performed and reported by a consultant radiologist. It revealed facial swelling and associated enhancement of the skin and subcutaneous tissue overlying the right side of the nose, nasolabial fold, and right cheek. The swelling extended superiorly to the medial aspect of the right cheek and inferiorly to the upper lip. No discrete rim-enhancing collections were observed. The orbit and conal spaces were clear. Complete opacification of the right maxillary sinus with a small isodense mucosal nodule in the posterior-inferior aspect of the sinus was observed.

**Figure 1:**
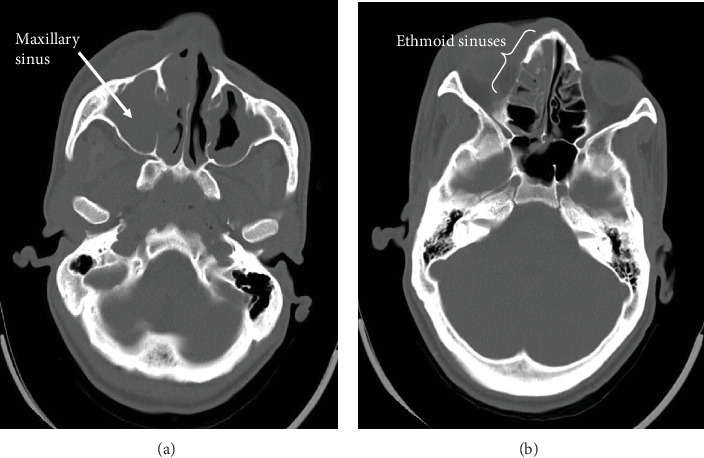
Axial slices of postcontrast CT of the brain and sinuses viewed on bony window: complete opacification of the right (a) maxillary sinus, with associated opacification of the anterior and middle (b) ethmoid sinuses. No bony erosion or sclerotic changes were observed.

**Figure 2:**
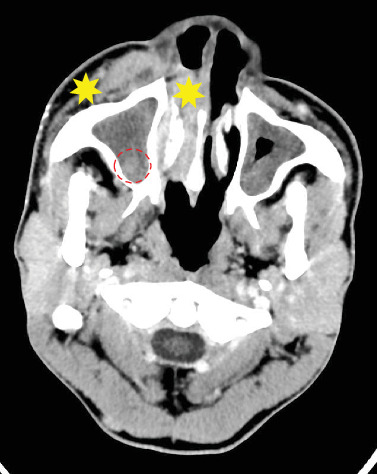
Axial slice of postcontrast CT of the brain and sinuses viewed on soft tissue window, showing facial swelling and postcontrast enhancement of the skin and subcutaneous tissue (yellow star) overlying the right side of the nose, nasolabial fold, and right cheek. An isodense mucosal nodule was observed in the posterior–inferior aspect of the sinus thought to be a polyp (red circle), with postcontrast enhancement and swelling of the inferior and middle nasal turbinates.

**Figure 3:**
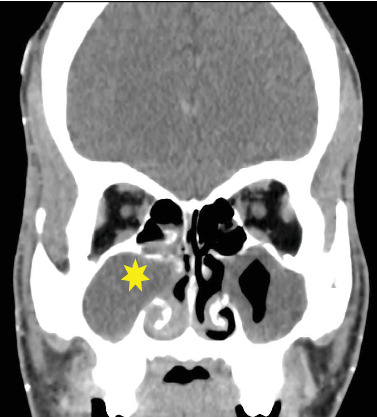
Coronal slice of postcontrast CT of the brain and sinuses viewed on soft tissue window, showing complete opacification (yellow star) of the right maxillary sinus, with widening of the osteomeatal complex.

Enhancement and swelling of the inferior and middle nasal turbinates were clearly demonstrated. No bony erosion or sclerotic changes were seen in the sinus walls. Circumferential mucosal thickening was present in the left maxillary sinus but with no enhancement, air fluid level, or bony changes. Opacification of the anterior and middle ethmoid sinuses was noted. No intracranial extra-axial collection or parenchymal changes were observed. The ventricular system, basal ganglia, and cerebellum were normal. Based on these observations, the radiologist reported an acute right maxillary sinusitis most likely from chronic osteomeatal complex obstruction, possibly due to a polyp, and facial cellulitis with no intracranial extension. The CT images are presented in Figures 1, 2 and 3.

### 2.5. Microbiological Results

Blood cultures were negative after 5 days of incubation in an automated instrument. Three tissue samples were submitted for tuberculosis (TB) work-up, bacterial microscopy, culture and sensitivity testing (MCS), and fungal cultures. All three samples had negative auramine stains, negative GeneXpert results, and negative TB culture results.

All three of the tissue samples cultured an *Acinetobacter* sp. One sample also cultured *Streptococcus parasanguinis*. These organisms were considered unlikely primary pathogens. Fungal cultures were positive for mold growth on one of the tissue samples. The laboratory was unable to identify the mold by phenotypic means and referred the cultured isolate to the National Health Laboratory Service (NHLS) Mycology Reference Laboratory for molecular testing. A pan-fungal PCR targeting the *ITS* gene was performed, followed by sequencing. The mold was identified as *S. oblongispora*.

### 2.6. Histology Results

Three specimens were also submitted for histopathological examination. The specimens were marked “nose biopsy,” “palate biopsy,” and “cheek biopsy,” respectively.

**Figure 4:**
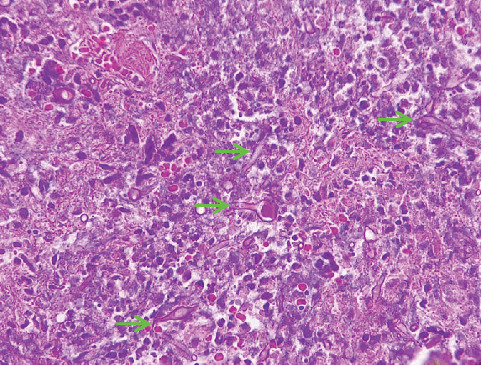
Hematoxylin and eosin (H&E) stain of a nasal biopsy received from a 32-year-old patient with a CD4 count of less than 50 cells/*μ*L. Numerous broad, aseptate fungal hyphae are seen (arrows) (200x magnification).

**Figure 5:**
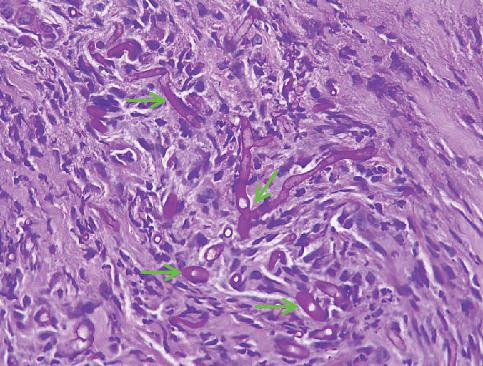
Periodic acid–Schiff (PAS) histochemical stain highlighting broad, ribbon-like, branching, aseptate fungal hyphae (arrows) (400x magnification).

**Figure 6:**
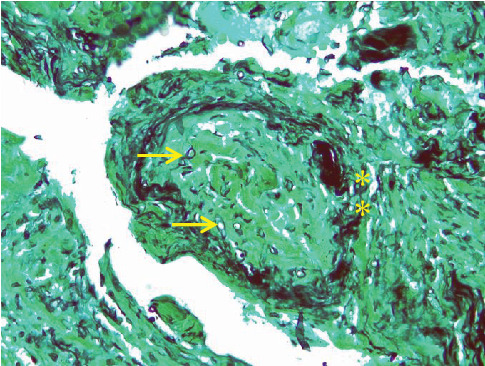
Grocott methenamine silver histochemical stain showing angioinvasive fungi both within the blood vessel lumen (arrows) and the vascular wall (asterisks) (400x magnification).

Microscopic examination of the tissue from the nose and cheek showed squamous epithelium, granulation tissue, active inflammation, and scattered thick-walled fungal elements with evidence of angioinvasion (Figure 4). The presence of fungi was confirmed by histochemical stains for fungal organisms, including periodic acid–Schiff (PAS) (Figure 5) and Grocott methenamine silver (Figure 6) stains. Necrotic bone spicules were also present. Microscopic examination of the palatal tissue revealed no abnormalities.

## 3. Discussion

To the best of our knowledge, this is the first case of *S. oblongispora* infection described in sub-Saharan Africa. It is also the first case described in the setting of HIV infection. This may be attributed to the underdiagnosis of fungal infections, potentially resulting from diagnostic challenges, rapid disease progression, and a high mortality rate.

*Saksenaea* spp., a filamentous mold belonging to the order Mucorales, are an uncommon cause of infection worldwide. *Saksenaea* spp. were first described in 1953 as *Saksenaea vasiformis* after isolation from soil in India [2, 3]. Subsequently, *Saksenaea* spp. have shown a rising incidence in human cases, responsible for diverse clinical manifestations mostly in tropical and subtropical regions [2, 3].

A recent systematic review reported that most cases were noted in India (27.7%), Australia (20%), the United States (12.3%), and Thailand (7.7%) [2]. With the improvement of fungal diagnostics and advancements in molecular techniques, a range of species have been recognized within the genus, including *S. vasiformis*, *Saksenaea erythrospora*, *S. oblongispora*, *Saksenaea loutrophoriformis*, *Saksenaea trapezispora*, and *Saksenaea. dorisiae* [1, 2].

Infection by this organism accounts for roughly 3% of human mucormycosis cases; the first being described by Ajello et al. in 1976 [2, 5]. As opposed to other causes of mucormycosis, *Saksenaea* spp. infections most frequently cause soft tissue infections in immunocompetent hosts following traumatic inoculation [2–5]. Rhinosinusitis and disseminated disease are uncommon but have been associated with high morbidity and mortality of up to 40% [2, 3, 5].

Cultivation of *Saksenaea* spp. is often possible using different culture media. However, it does not sporulate easily, making phenotypic identification difficult [2, 5]. In our case, Sabouraud dextrose media was used and incubated at 26°C and 35°C, respectively, according to the local laboratory's standard operating procedures. A study from Spain reported that in all reported cases where Sabouraud dextrose media was used, fungal growth was achieved, but it was not possible in any of the cases to achieve sporulation of the fungus to identify it phenotypically [5]. Therefore, it was no surprise that the laboratory was unable to identify the organism phenotypically and required molecular diagnostics for identification.

Histologic evaluation is often the mainstay of the diagnosis of mucormycosis [1]. Fungal cultures have suboptimal sensitivity and often require extensive periods of incubation for the pathogens to grow and be identified [1, 6]. It has been advised to combine different testing platforms, including at least microbiology and histopathology, to optimize the detection of invasive fungal infections [1].

Imaging plays an important role in the early detection of invasive fungal infections, guiding prompt antifungal treatment, and evaluating response to therapy. MRI and conventional CT are the modalities of choice. While imaging cannot definitively identify the fungal pathogen or even confirm a fungal cause, it can reveal patterns of types of infections and the extent of involvement that help direct appropriate biopsy sites [7].

Due to the limited available data on infections caused by *S. oblongispora*, there are no definitive guidelines regarding the most effective antifungal agents [2]. The treatment of mucormycosis usually requires immediate, aggressive surgical debridement combined with appropriate antifungal therapy, primarily intravenous amphotericin B (AMB), either as monotherapy or in combination with posaconazole [1, 2, 5]. Badali et al. [8] found that AMB exhibited the strongest in vitro activity against *Saksenaea* species isolated from clinical cases in the United States, with all isolates showing minimum inhibitory concentrations (MICs) of ≤ 0.3 *μ*g/mL. Among the azoles, posaconazole demonstrated the greatest potency, with MICs between 0.06 and 0.125 *μ*g/mL, followed by isavuconazole, which showed MICs ranging from 0.25 to 2 *μ*g/mL. In contrast, itraconazole displayed considerable variability, with MICs ranging from 0.125 to over 16 *μ*g/mL [8].

An invasive fungal infection was not suspected in this patient. He received neither antifungal therapy nor surgical interventions, and unfortunately, he died. Although the patient also had advanced HIV infection with a low CD4 count, his clinical features attributed to this invasive fungal infection were pronounced. There was an expeditious deterioration of his symptoms during admission, suggesting that this IFI was the likely cause of death.

The mortality rate of mucormycosis reported in a recent meta-analysis was 36.9% [2]. A noteworthy finding was that underlying comorbidities such as hypertension and being immunocompromised were not associated with severe outcomes. Hospital-associated wounds were significantly linked to increased mortality [2].

## 4. Conclusion

Mucormycosis is a life-threatening fungal infection with significant mortality. A high index of suspicion is required to initiate aggressive, appropriate therapy timeously. A multidisciplinary diagnostic and management approach is important and ideally consists of infectious disease physicians and experts in the field of otolaryngology, microbiology, histopathology, and, if available, pharmacology to assist with therapeutic drug monitoring. Collaborating across disciplines enables optimal patient care and outcomes, which is not always possible in resource-constrained areas. However, even with rapid, accurate evaluation, diagnosis, and treatment by a multidisciplinary team, the prognosis of mucormycosis remains poor.

## Data Availability

The data that support the findings of this study are available from the corresponding author upon reasonable request.
